# Structural lesions and transcriptomic specializations shape gradient perturbations in Wilson disease

**DOI:** 10.1093/braincomms/fcae329

**Published:** 2024-09-24

**Authors:** Sheng Hu, Chuanfu Li, Yanming Wang, Taohua Wei, Xiaoxiao Wang, Ting Dong, Yulong Yang, Yufeng Ding, Bensheng Qiu, Wenming Yang

**Affiliations:** Department of Electronic Engineering and Information Science, Medical Imaging Center, University of Science and Technology of China, Hefei, Anhui, 230026, China; Institute of Advanced Technology, University of Science and Technology of China, Hefei, Anhui, 230094, China; School of Medical Information Engineering, Anhui University of Traditional Chinese Medicine, Hefei, Anhui, 230012, China; Medical Imaging Center, First Affiliated Hospital of Anhui University of Traditional Chinese Medicine, Hefei, Anhui, 230031, China; Department of Electronic Engineering and Information Science, Medical Imaging Center, University of Science and Technology of China, Hefei, Anhui, 230026, China; Department of Neurology, First Affiliated Hospital of Anhui University of Traditional Chinese Medicine, Hefei, Anhui, 230031, China; Key Laboratory of Xinan Medicine of the Ministry of Education, Anhui University of Traditional Chinese Medicine, Hefei, Anhui, 230031, China; Department of Electronic Engineering and Information Science, Medical Imaging Center, University of Science and Technology of China, Hefei, Anhui, 230026, China; Department of Neurology, First Affiliated Hospital of Anhui University of Traditional Chinese Medicine, Hefei, Anhui, 230031, China; Key Laboratory of Xinan Medicine of the Ministry of Education, Anhui University of Traditional Chinese Medicine, Hefei, Anhui, 230031, China; Department of Neurology, First Affiliated Hospital of Anhui University of Traditional Chinese Medicine, Hefei, Anhui, 230031, China; Key Laboratory of Xinan Medicine of the Ministry of Education, Anhui University of Traditional Chinese Medicine, Hefei, Anhui, 230031, China; Department of Neurology, First Affiliated Hospital of Anhui University of Traditional Chinese Medicine, Hefei, Anhui, 230031, China; Key Laboratory of Xinan Medicine of the Ministry of Education, Anhui University of Traditional Chinese Medicine, Hefei, Anhui, 230031, China; Department of Electronic Engineering and Information Science, Medical Imaging Center, University of Science and Technology of China, Hefei, Anhui, 230026, China; Institute of Advanced Technology, University of Science and Technology of China, Hefei, Anhui, 230094, China; Department of Neurology, First Affiliated Hospital of Anhui University of Traditional Chinese Medicine, Hefei, Anhui, 230031, China; Key Laboratory of Xinan Medicine of the Ministry of Education, Anhui University of Traditional Chinese Medicine, Hefei, Anhui, 230031, China

**Keywords:** Wilson disease, functional gradient, gene expression, gray matter volume, transcriptomic specialization

## Abstract

Functional dysregulations in multiple regions are caused by excessive copper deposition in the brain in Wilson disease (WD) patients. The genetic mechanism of WD is thought to involve the abnormal expression of *ATP7B* in the liver, whereas the biological and molecular processes involved in functional dysregulation within the brain remain unexplored. The objective of this study was to unravel the underpinnings of functional gradient perturbations underlying structural lesions and transcriptomic specializations in WD. In this study, we included 105 WD patients and 93 healthy controls who underwent structural and functional MRI assessments. We used the diffusion mapping embedding model to derive the functional connectome gradient and further employed gray matter volume to uncover structure–function decoupling for WD. Then, we used Neurosynth, clinical data, and whole-brain gene expression data to examine the meta-analytic cognitive function, clinical phenotypes, and transcriptomic specializations related to WD gradient alterations. Compared with controls, WD patients exhibited global topographic changes in the principal pramary-to-transmodal gradient. Meta-analytic terms and clinical characteristics were correlated with these gradient alterations in motor-related processing, higher-order cognition, neurological symptoms, and age. Spatial correlations revealed structure–function decoupling in multiple networks, especially in subcortical and visual networks. Within the cortex, the spatial association between gradient alterations and gene expression profiles has revealed transcriptomic specilizations in WD that display properties indicative of ion homeostasis, neural development, and motor control. Furthermore, for the first time, we characterized the role of the *ATP7B* gene in impacting subcortical function. The transcriptomic specializations of WD were also associated with other neurological and psychiatric disorders. Finally, we revealed that structural lesions and gradient perturbations may share similar transcriptomic specializations in WD. In conclusion, these findings bridged functional gradient perturbations to structural lesions and gene expression profiles in WD patients, possibly promoting our understanding of the neurobiological mechanisms underlying the emergence of complex neurological and psychiatric phenotypes.

## Teaser

Hu et al. report functional gradient perturbation in Wilson disease. Association analysis between gradient perturbation and transcriptional data reveals that the ATP7B gene is for the first time identified to impact on subcortical function. Further spatial correlation analysis suggests gradient perturbation sharing similar transcriptional specializations with structural lesions.

## Graphical Abstract

**Graphical Abstract:**
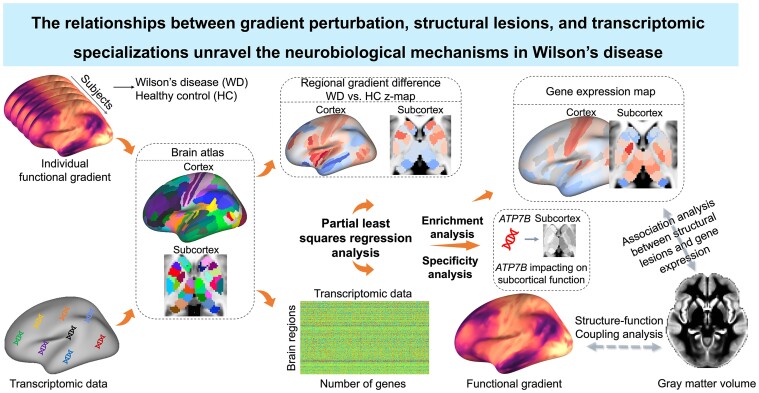

## Introduction

Wilson disease (WD) is an autosomal recessive disorder of copper metabolism caused by a lack of biliary copper excretion.^1^ Mutation of the *ATP7B* gene, which is most highly expressed in the liver, results in the absence and reduced expression of the *ATP7B* protein, identifying it as causative of copper dysmetabolism.^2^ Excessive copper accumulation in the liver and brain ultimately leads to neurological and psychiatric phenotypes in WD patients.^3^ However, in an animal model of WD, *ATP7B* is also expressed in neuronal cells of the hippocampus, olfactory bulbs, cerebellum, brainstem, and cerebral cortex. The concurrent high levels of copper in these regions further indicated that dysfunctional *ATP7B* protein is related to local copper accumulation and the cerebral manifestations of WD.^4^

The toxic effect of copper results in damaged astrocytes, neurons, and oligodendrocytes, which gradually develop structural lesions, especially in subcortical regions (SUB), including the basal ganglia, thalamus, and brainstem.^5,6^ Notably, multimodal MRI studies revealed functional abnormalities across the entire brain and structure–function decoupling in WD patients.^7,8^ Furthermore, defective cortical function may be due to dysfunction of cortio-subcortical pathways, leading to neurological and psychiatric symptoms in WD patients.^9,10^ However, the neurobiological mechanisms underlying brain dysfunction remain to be elucidated. Given that genes have an essential influence on brain function,^11^ brain dysfunction in WD may also be shaped by specific mutated genes, such as *ATP7B*. Moreover, considering the variability of gene expression profiles across the entire brain, such as the cortex and SUB,^12^ their relation to the WD topology of dysfunction may provide insights into the neurobiological pathways contributing to the neurological pathogenesis of WD.

Brain hierarchical theories revealed that functional connectome patterns are governed by the graded macroscale axis that spans the primary sensorimotor areas to transmodal regions of the default mode network (DMN).^13^ Perturbation in these patterns shapes clinical phenotypes, such as perception, cognition, and neurological symptoms, in multiple neurodegenerative diseases.^14,15^ Furthermore, the macroscale axis of the cortex is also intrinsic to gene expression and microstructural topography, indicating that the functional basis strongly depends on intact gene expression and microstructural profiles.^12^ Although many studies have revealed alterations in functional connectivity (FC) involving the basal ganglia, sensorimotor, and DMN regions in patients with WD,^7,8,16^ no studies have reported whether and how these alterations are affected by structural and transcriptomic vulnerability. Therefore, if WD patients exhibit perturbations in the functional hierarchy, these abnormalities might be associated with gene expression and structural topography. The illustration of such alterations would provide complementary insights into the structural and molecular genetic underpinnings of the dysfunctional hierarchy in WD.

Functional connectome patterns are facilitated by decomposing FC data into different gradients to capture the topography of the connectome.^17^ Such a principal functional gradient is present in the macroscale organization of functional brain networks, reflecting the hierarchical macroscale axis.^13^ This study aimed to investigate the structural and genetic underpinnings of functional hierarchical perturbation in WD. For this purpose, we mapped the functional gradient using functional MRI and determined the perturbation of the hierarchical macroscale axis of the WD. Then, the gray matter volume (GV) evaluated by 3D high-resolution MRI was used to perform a spatial correlation analysis, revealing whether structure–function is decoupled in WD. Moreover, we calculated the spatial correlation of the connectome gradient perturbation with whole-brain gene expression data from the Allen Human Brain Atlas^18^ (AHBA) and further examined whether structural lesions and gradient perturbations are mediated by similar transcriptomic specializations. Gene enrichment analysis was finally used to probe transcriptomic associations for known pathogenic WD variants, as well as other neurological and psychiatric disorders.

## Materials and methods

### Experimental design

This study combined resting-state fMRI and transcriptomic data to determine links between gene expression and perturbations in functional gradients in individuals with WD relative to healthy control (HC) and to identify whether structural lesions and gradient perturbations are mediated by similar transcriptomic specializations (Supplementary Fig. 1). We first used a large cohort of resting-state fMRI data to calculate the functional gradient, followed by assessing gray volume for all participants. Then, the spatial correlation between the GV and functional gradient was calculated to determine whether the structure–function relationship was decoupled in the WD. Changes in functional gradients were further associated with gene expression data using partial least squares (PLS) regression analysis, followed by gene enrichment and specificity analyses to evaluate the biological processing of the gradient perturbation. Finally, spatial correlation analysis was performed to assess whether the gradient perturbations shared similar transcriptomic specializations with structural lesions in WD.

### Participants

We studied a larger cohort of 105 WD patients (65 males, mean ± SD age = 27.29 ± 4.02 years) and 93 HCs (60 males, mean ± SD age = 24.48 ± 2.26 years) collected from the First Affiliated Hospital of Anhui University of Chinese Medicine. All patients met the diagnostic criteria, with a ceruloplasmin concentration < 0.1 g/L, a 24-h urinary copper excretion >100 μg, and the presence of a K-F ring on slit-lamp examination. Unified Wilson’s Disease Rating Scale neurological examination (UWDRS-N) subscores were recorded as a measure of neurological disorder severity.^19^ The exclusion criterion for WD patients was a diagnosis of other diseases. Normal individuals with a history of mental health problems or other serious diseases were excluded from this study. The Ethics Committee of the First Affiliated Hospital of Anhui University of Chinese Medicine gave ethical approval for this work, and written informed consent was obtained from all participants. Demographic and clinical information is described in Table 1.

**Table 1:** Demographic and clinical characteristics of the WD and HC

	WD	HC	*P*-value
	*n* = 105	*n* = 93	
Age mean (SD), years	27.23 ± 8.30	24.48 ± 2.26	0.0023
Males, *n* (%)	59 (60%)	60 (64%)	-
Education mean (SD), years	11.97 ± 3.39	15.42 ± 4.06	<0.001
Disease duration mean (SD), year	9.13 ± 6.67	-	
UWDRS-N score mean (SD)	10.06 ± 12.64	-	-
Ceruloplasmin mean (SD), g/L	0.041 ± 0.04	-	-
24 h urinary cooper excretion mean (SD), μg	850.85 ± 588.56	-	-

HC, healthy control; WD, Wilson’s disease.

### Data acquisition and preprocessing

The data were obtained at the First Affiliated Hospital of Anhui University of Chinese Medicine using a GE MR750 scanner. Resting-state functional magnetic resonance (R-fMRI) data were acquired using a gradient-echo single-shot echo-planar imaging sequence with the following parameter settings: repetition time, 2000 ms; echo time, 30 ms; flip angle, 90°; matrix size, 64 × 64; field of view, 220 mm × 220 mm; resolution of the axial slice, 3.4375 × 3.4375 mm^2^; and slice thickness, 3 mm. A total of 185 volumes were acquired from each individual, with 36 slices per volume. T1-weighted images were collected using a T1-3D BRAVO sequence: repetition time, 8.16 ms; echo time, 3.18 ms; flip angle, 12°; matrix size, 256 × 256; field of view, 256 mm × 256 mm; voxel size, 1 mm^3^; and slice thickness, 1 mm. R-fMRI data were preprocessed using a standard pipeline, including slice timing, head motion correction, normalization, and bandpass, as described in the Supplementary Materials.

### GV estimation

GV measurements were performed using a voxel-based morphometric (VBM) approach^20^ that is embedded in the FSL-VBM toolbox. Briefly, the gray matter (GM) was first segmented from the T1-weighted images, followed by normalization to the MNI152 standard space. The normalized images were further applied to create a study-specific GM template. All native GM images were subsequently registered to the template and modulated for contraction. The modulated GM images were finally smoothed using an isotropic Gaussian kernel with a sigma of 3 mm.

### Preprocessing gene expression data

The microarray gene expression data were obtained from six donors (mean age: 42.5 years, 5 males and 1 female), including two complete brains and four left hemispheres (http://human.brain-map.org/).^21^ Here, we used the gene data of two male donors with complete brain in this study. None of the donors had a known history of neuropsychiatric or neurological conditions. The gene expression of each sample from all donors was quantified across 58 692 probes, resulting in each sample containing expression levels of 20 737 genes. The tissue samples were also spatially registered to the Montreal Neurological Institute (MNI) coordinate space, and the locations of each sample were recorded with MNI coordinates.^21^ We conducted gene expression analysis within the cortical and SUB due to the large transcriptional differences between the two main regions.^22^ The standard preprocessing pipeline for the gene expression microarray data used in the present study included mapping the tissue samples onto a cortical parcellation with 360 parcels^23^ and a subcortical parcellation with 54 parcels,^24^ probe reannotation and selection, and normalization across donors.^18^

### Functional connectome gradient analysis

We constructed individual functional connectomes at the voxel level. To reduce the computational complexity, we resampled the preprocessed R-fMRI data to a 4-mm isotropic resolution. For each individual, a FC matrix was first constructed by performing Pearson correlation analysis between each pair of GM nodes (20 208 voxels). Then, the FC matrix was further subjected to diffusion map embedding to calculate the connectome gradient.^17,25^ Specifically, the FC matrix was thresholded to retain the top 10% connections of each node, and the cosine similarity between each pair of nodes was computed. Furthermore, the similarity matrix was scaled into a normalized angle matrix to avoid negative values.^26,27^ The diffusion map embedding approach was finally applied to identify gradient components that explain most functional connectome variances. Following the previous recommendation, we set the manifold learning parameter *α* = 0.5 in the diffusion process.^27^ Given that the principal gradient to an extreme represents the macroscale axis, we focused primarily on perturbation in the principal gradients of WD.^13^ The case–control differences between WD patients and HCs in the connectome gradient were evaluated by a two-sample *t* test with age, sex, and education as covariates. The false discovery rate (FDR) was used to achieve multiple comparison corrections at the voxel level. The statistically significant threshold was set to *q* < 0.05. In addition, the whole-brain voxels were assigned to eight systems according to a cortical atlas with seven parcels and a Harvard-Oxford probabilistic subcortical atlas. The system-based differences in the connectome gradient between WD patients and HCs were further examined using a paired two-sample *t* test with age, sex, and education as covariates. The findings were further corrected by multiple comparisons using an FDR of 0.05.

### Association analysis between meta-analytic cognitive terms and gradient changes in WD

Neuosynth (https://neurosynth.org/)^28^ was used to evaluate the relationships between the meta-analytic cognitive terms and functional gradient alterations in WD patients. The thresholded Z-map derived from the between-group comparisons of the gradient was first divided into WD-positive (WD > HC) and WD-negative (WD < HC) maps. The ‘decoder’ function in Neurosynth was used to identify the spatial correlations between each map and the meta-analytic map of each term in the database.^29^ Finally, the top 30 cognitive terms were selected.

### Correlation analysis between clinical characteristics and gradients in WD patients

We examined the relationships between gradients in perturbed regions and clinical characteristics, such as UWDRS-N score, age, ceruloplasmin concentration, and 24-h urinary copper excretion, using partial correlations controlled for age and sex.

### Association analysis between the GV and gradients

To investigate whether structure–function is decoupled in WD, the spatial association between averaged GV and averaged functional connectome gradients was estimated using spatial correlation analysis. Spatial correlation analyses were performed based on the whole brain and eight systems. For all spatial correlation analyses, findings were corrected using permutation tests (*N* = 10 000) at *P* = 0.05. The results were further corrected for multiple comparisons using an FDR of 0.05. The spatial correlation was also calculated at the subject level. Then, the differences in spatial correlation between WD patients and HCs across subjects were assessed using a two-sample *t* test, and the results were corrected by an FDR of 0.05. Finally, using a nonparametric bootstrapping approach (10 000 times), we performed a mediation analysis in which the altered gradient was taken as an independent variable, GV in altered gradient regions was taken as the mediator, and UWDRS-N was taken as the dependent variable.

### Association analysis between gene expression and WD gradient changes

The spatial correlations between principal gradient changes and gene expression were examined in cortical and SUB. Given the high dimensionality of AHBA data, we used PLS regression,^30^ a multivariate linear model, to reveal the group of weighted genes (or PLS components) that best explained the differences in the gradient. Briefly, we first aligned the gene expression data (10 027 genes) and between-group difference Z-maps of the principal gradient to a cortical atlas^23^ and subcortical atlas.^24^ In PLS analysis, the gene expression data were used to identify the linear combinations of genes that best predicted response variables^31^ (Z-map of between-group differences of the principal gradient). The statistical significance of the first two PLS components was tested based on 10 000 permutations of the response variables. A bootstrapping approach was finally used to correct the estimation error of the weight of each gene on each PLS component.^31^

### Enrichment analysis

We first ranked the genes according to their bootstrap weights (absolute values). Then, the top 10th percentile of 10 027 genes was applied to a web-based gene set analysis toolkit^32^ (https://www.webgestalt.org/) to uncover biological processes enriched in the list of genes. The enrichment ratio (ER) was calculated as the number of PLS-derived genes overlapping with each biological process or molecular function divided by the number of genes expected to overlap according to random permutations. Significant enrichment was determined with Bonferroni FDR-corrected *q* < 0.05.

### Specificity analysis

Specificity analysis was used to assess whether known genes (*ATP7B*) causing WD were enriched in the PLS components. We also performed specificity analysis to uncover the enrichment of other neurological and psychiatric risk genes in the PLS components. The disorder-related risk genes, provided by the AHBA (https://help.brain-map.org/display/humanbrain/Documentation), are listed in Supplementary Table 1.

We calculated the ER for each PLS component. The ER is defined as the difference between the mean bootstrap weight of the candidate gene and the mean bootstrap weight of the same number of randomly permuted genes, which was further divided by the standard deviation weight of the permuted genes.^33^ Significance was determined by the percentile of the bootstrap weight of the candidate genes relative to the bootstrap weights of randomly selected genes from 10 000 permutations. Positive/negative ER of a given condition indicates that the risk genes are expressed to a higher/lower degree relative to the baseline expression level.

### Association analysis between structural changes and PLS components

To investigate whether structural changes and disturbances in the connectome gradient shared similar transcriptomic specializations, we calculated the spatial correlations between structural changes and each PLS component in WD. We applied a threshold of 0 to |0.1| for the PLS-score in the spatial correlation analysis. Structural changes were defined as the absolute value of the between-group difference in GV in high (the PLS-score was a threshold of 0 to 0.1 with a step of 0.01) or low (the PLS-score was a threshold of −0.1 to 0 with a step of 0.01) gene expression regions. The spatial correlations were corrected for spatial autocorrelations using a permutation test (*N* = 10 000).

### Statistical analyses

The case–control differences between WD patients and HCs in the connectome gradient were evaluated by a two-sample *t* test with age, sex, and education as covariates. The system-based differences in the connectome gradient between WD patients and HCs were further examined using a paired two-sample *t* test with age, sex, and education as covariates. The relationships between GV and the functional gradient were evaluated via spatial correlation analysis, and the statistical significance was evaluated via permutation tests (*N* = 10 000). The differences in the spatial correlation of GV and functional gradient between WD patients and HCs across subjects were assessed using a two-sample *t* test. The relationships between structural changes and each PLS component in the WD were evaluated via spatial correlation analysis, and the statistical significance was evaluated via permutation tests (*N* = 10 000). In the specificity analysis, the statistical significance of ER was assessed using a bootstrapped permutation test (*N* = 10 000). A two-tailed *P* < 0.05 after FDR correction for multiple comparisons or correlations was considered significant for all tests. GV differences between WD patients and HCs were evaluated using permutation-based nonparametric testing with age, sex, and education as covariates. The statistical significance was set as *P* < 0.001, using a threshold-free cluster enhancement approach with familywise error correction for multiple comparisons.^34^

## Results

### Demographics

Table 1 compares demographic and clinical variables between the WD patients and HCs. On average, the subjects with WD were older than the HCs were. As expected, the years of education were significantly different between the WD patients and HCs.

### Perturbation of the primary-to-transmodal gradient in the WD

**Figure 1:**
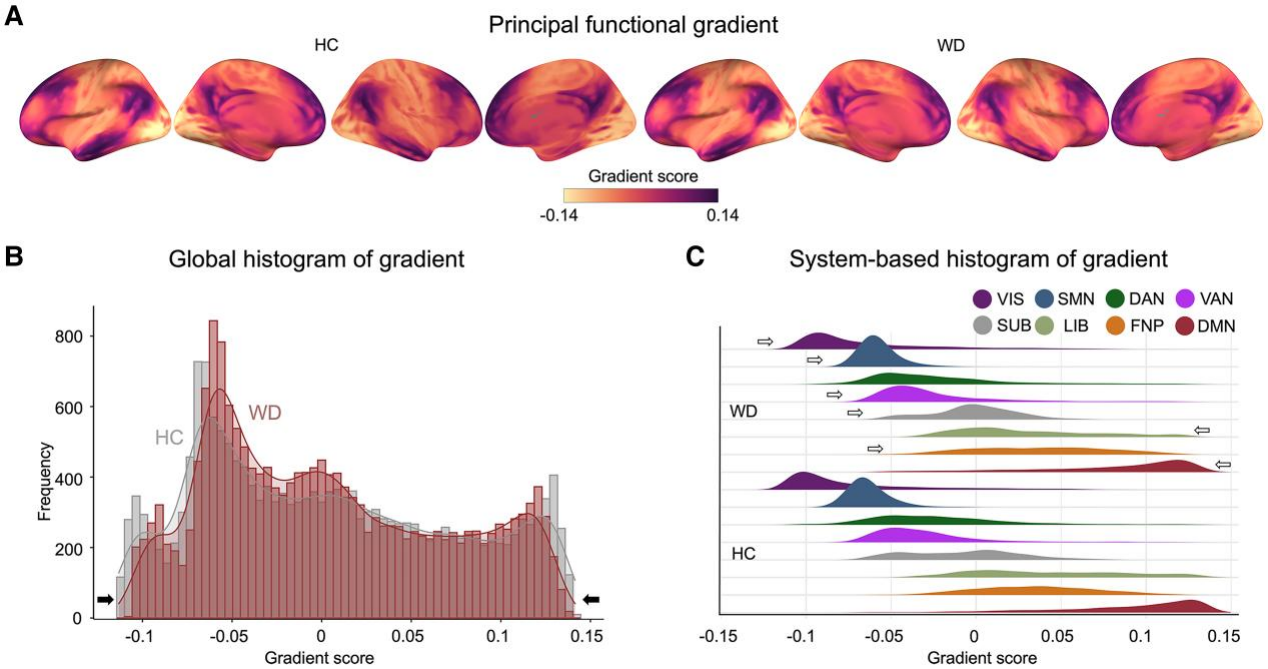
**Connectome gradient mapping in WD patients and HCs.** The group-level connectome gradients of both WDs (*n* = 105) and HCs (*n* = 93) were calculated from the group mean FC matrix. (**A**) The principal gradient was organized along a gradual axis from the primary visual/sensorimotor networks to the DMN. (**B**) Global histogram of the gradient. The extreme gradient values were suppressed in the WD patients relative to those in the HCs. (**C**) System-based histogram of the gradient. The system-based differences in the connectome gradient between WD patients and HCs were further examined using a paired two-sample *t* test with age, sex, and education as covariates. Arrows indicate the direction of the significant differences between the WD and HC groups. The system-based differences in the principal gradient distribution between WD patients and HCs were evaluated using two-sample *t* tests with age, sex, and education as covariates. A two-tailed *P* < 0.05 after FDR correction for multiple comparisons was considered significant. DAN, dorsal attention network; DMN, default mode network; FPN, frontoparietal network; HC, healthy control; LIB, limbic network; SMN, sensorimotor network; SUB, subcortical regions; VAN, ventral attention network; VIS, visual network; WD, Wilson’s disease.

The principal gradient explained 12.2 ± 1.8% of the total variance in the connectome across all individuals (WD: 11.8 ± 1.7%, HC: 12.9 ± 1.8%, Supplementary Fig. 2), which was organized along a graded macroscale axis from the primary visual/sensorimotor (VIS/SMN) areas to the DMN (Fig. 1A), consistent with previous observations of connectome gradients from primary to transmodal regions in the healthy brain.^17^ The spatial patterns of the principal gradient maps were highly similar between the WD and HC groups (Spearman’s *R* = 0.9734, *P* < 0.0001, spatial autocorrelation corrected by permutation tests; Supplementary Fig. 3). The global histogram confirmed that the extreme values were suppressed in WD patients relative to HCs, while those in the mid-range increased (Fig. 1B). For each system, we further computed the gradient score differences between group-averaged maps of WD patients and HCs by using paired *t* tests across voxels. The WD group had a greater gradient score in the VIS, SMN, ventral attention network (VAN), frontoparietal network (FPN), and SUB but a lower gradient score in the limbic network (LIB) and DMN (FDR-corrected *q* < 0.05, Fig. 1C and Supplementary Table 2). The results of the secondary gradient are provided in Supplementary Fig. 4 and Supplementary Table 4.

### Changes in the connectome gradient in WD

**Figure 2:**
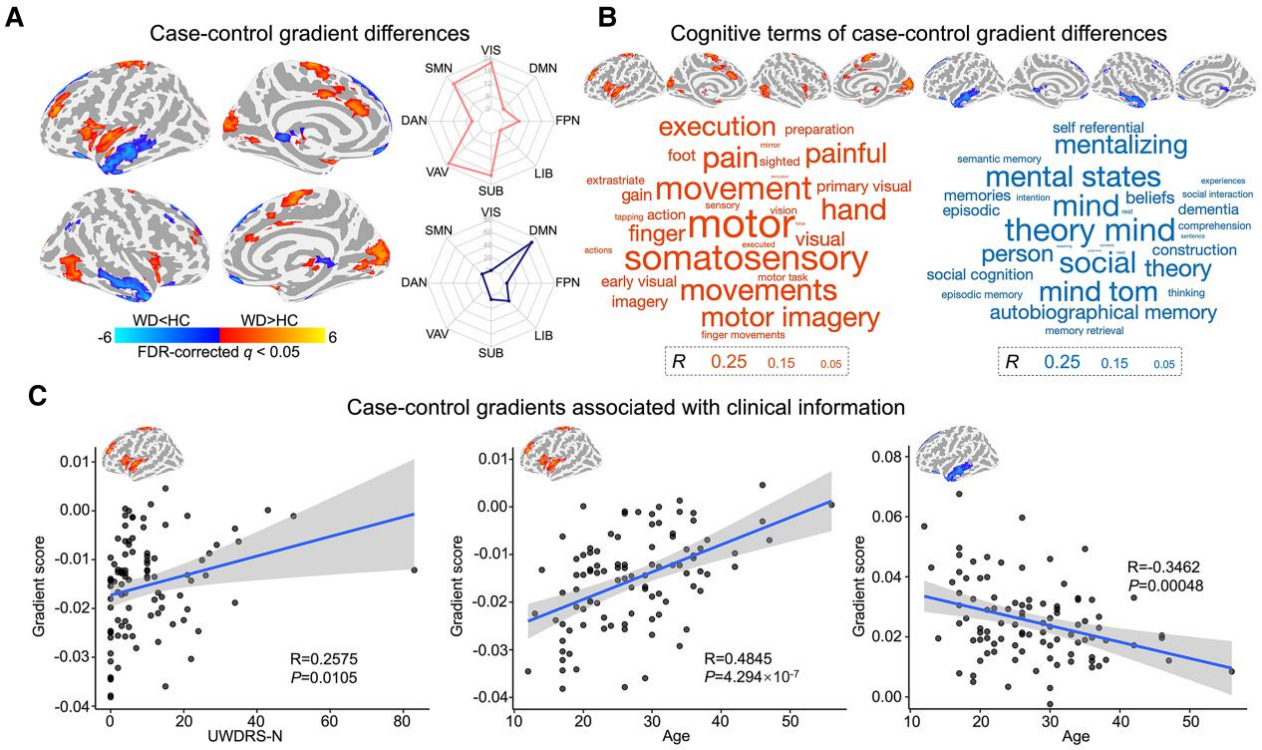
**Gradient differences and their associations with cognitive function.** (**A**) Voxel wise statistical comparisons between WD patients and HCs and distribution of regional case–control differences in different systems. Higher/lower values in WD are presented as warm/cold colors. Two-sample *t* test was performed to evaluate group differences, with age, sex, and education as covariates. The statistical significance level was set as FDR voxel level-corrected *q* < 0.05. (**B**) Word clouds of cognitive functions associated with brain regions that exhibited higher or lower gradient scores in WD. The font size of a given cognitive term corresponds to the correlation coefficient of the between-group Z-map of the principal gradient with the meta-analytic map of that term generated by Neurosynth. (**C**) Associations between regional gradient scores and individual UWDRS-N scores and age. The projection of each point on the vertical and horizontal coordinates represents the mean principal gradient score within the region with significant case–control differences and clinical characteristics, such as the UWDRS-N score and age. DAN, dorsal attention network; DMN, default mode network; FPN, frontoparietal network; HC, healthy control; LIB, limbic network; SMN, sensorimotor network; SUB, subcortical regions; VAN, ventral attention network; VIS, visual network; WD, Wilson’s disease.

The WD group showed lower gradient scores mostly in the DMN [medial temporal pole (mTP) and medial prefrontal cortex (mPFC), 70.3%] and LIB (19.4%) but higher scores mainly in the VIS [primary visual cortex (V1), 22.8%], SMN [supplementary motor areas (SMA), 19.9%], VAN (insula, 23.3%) and SUB (striatum, thalamus, 20.6%) than did the HC group (absolute *d* = 0.08–0.19, FDR-corrected *q* < 0.05, Fig. 2A, Supplementary Table 3). For secondary connectome gradients, only one region showed a significantly greater gradient score in the WD group than in the HC group (Supplementary Fig. 5 and Supplementary Table 5).

### Gradient changes associated with cognitive functions in WD patients

Higher principal gradient scores in the WD were mainly involved in motor-related processes, such as movement, motor imagery, and somatosensory processes (Fig. 2B and Supplementary Table 6). Lower principal gradient scores in the WD group were correlated with higher-order cognitive processes, including mental states, mentalizing, theory of mind, and social factors (Fig. 2B and Supplementary Table 6).

### Changes in principal gradients correlate with WD-specific clinical characteristics

The regions with significantly greater gradient scores in WD were positively correlated with UWDRS-N (*R* = 0.2575, *P* = 0.0105, Fig. 2C) and age (*R* = 0.4845, *P* = 4.294 × 10^−7^, Fig. 2C). The regions with significantly lower gradient scores in WD were negatively correlated with age (*R* = −0.3462, *P* = 0.00048; Fig. 2C). No significant correlations were detected between the gradient scores and the ceruloplasmin concentration, 24-h urinary copper excretion, or disease duration.

### GV and connectome gradient decoupling in WD

By evaluating group differences in GV between WD patients and HCs, we found that GV was significantly reduced in the SMN, VIS, DMN, and SUB in WD patients (Supplementary Fig. 6 and Supplementary Table 7). Conversely, significant increases in GV were detected in the VAN, dorsal attention network, LIB, and FPN in WD (Supplementary Fig. 6 and Supplementary Table 7).

**Figure 3:**
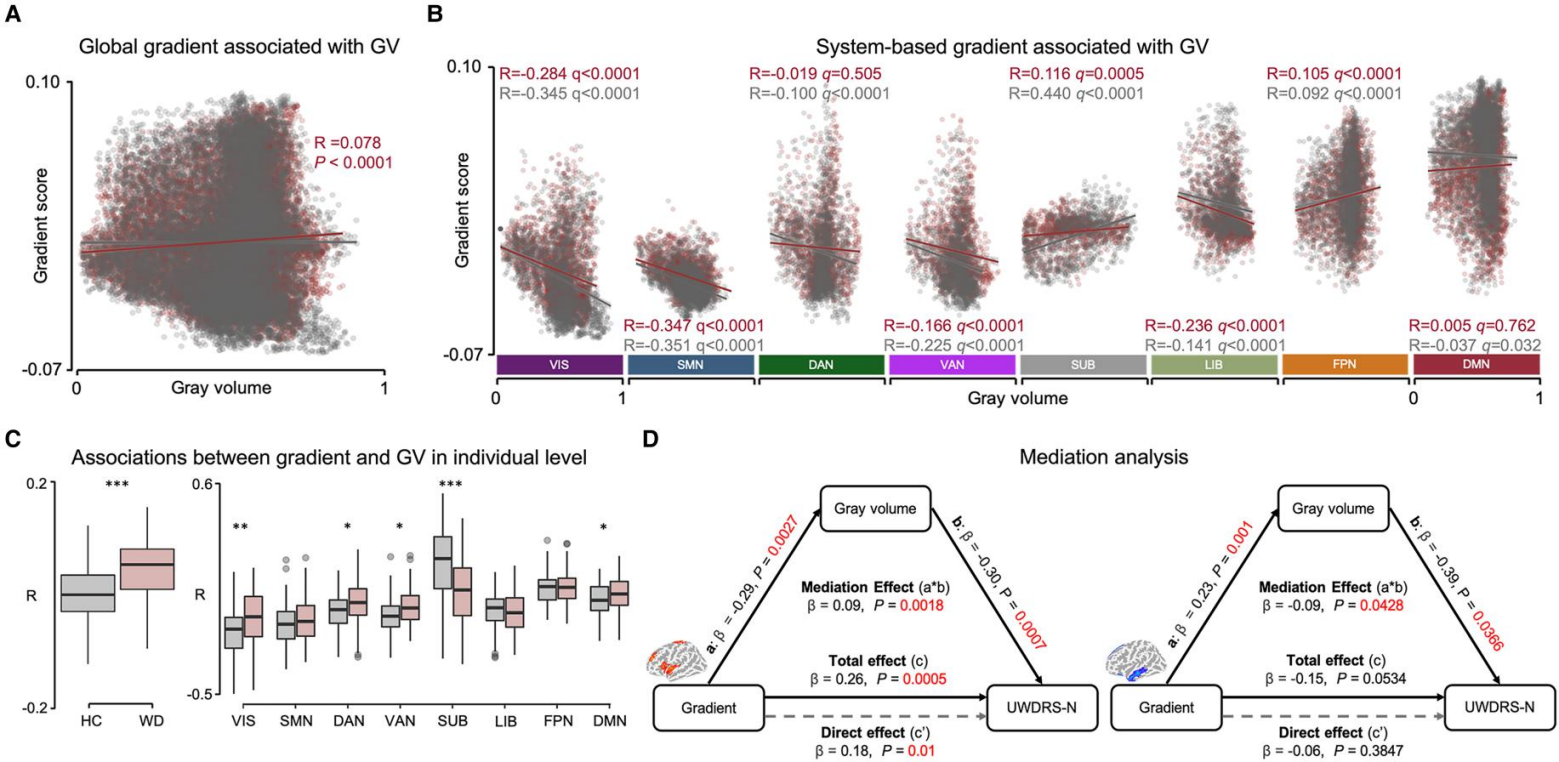
**GV and principal gradient decoupling in WD. (A) Global association between gradient and GV in WD patients and HCs.** The projection of each point on the vertical and horizontal coordinates represents the principal gradient score and gray volume value, respectively, in a given voxel. (**B**) System-based associations between gradient and GV in WD patients and HCs. The *P* values were corrected by FDR. The projection of each point on the vertical and horizontal coordinates represents the principal gradient score and gray volume value, respectively, in a given voxel. (**C**) Global and system-based associations between gradient and GV at the individual level and their group differences between WD patients (*n* = 105) and HCs (*n* = 93) were measured by a two-sample *t* test and corrected by FDR. (**D**) GV significantly mediated the associations of regional gradients with the UWDRS-N score. We performed a mediation analysis in which the altered gradient was taken as an independent variable, GV in altered gradient regions was taken as the mediator, and UWDRS-N was taken as the dependent variable. The statistical significance was performed using a nonparametric bootstrapping approach (10 000 times). *P < 0.05; **P < 0.01; ***P < 0.001. HC, healthy control; WD, Wilson’s disease.

High spatial correlations between the principal gradient and GV were observed in the VIS, SMN, VAN, SUB, and LIB (Fig. 3B) in both the WD and HC groups but not in the whole brain (Fig. 3A). At the subject level, the spatial correlation was significantly different between the two groups in the extensive networks, especially in the VIS and SUB (Fig. 3C), indicating function–structure decoupling in these regions. The spatial correlation between the secondary gradient and GV is shown in Supplementary Fig. 7.

We also found that the GV in regions with higher (meditation effect: β=0.09,P=0.0018) and lower (meditation effect: β=−0.09,P=0.0428) gradient scores in WD partially mediated the relationship between the gradient and UWDRS-N (Fig. 3D).

### Changes in gene expression associated with WD-related gradients

**Figure 4:**
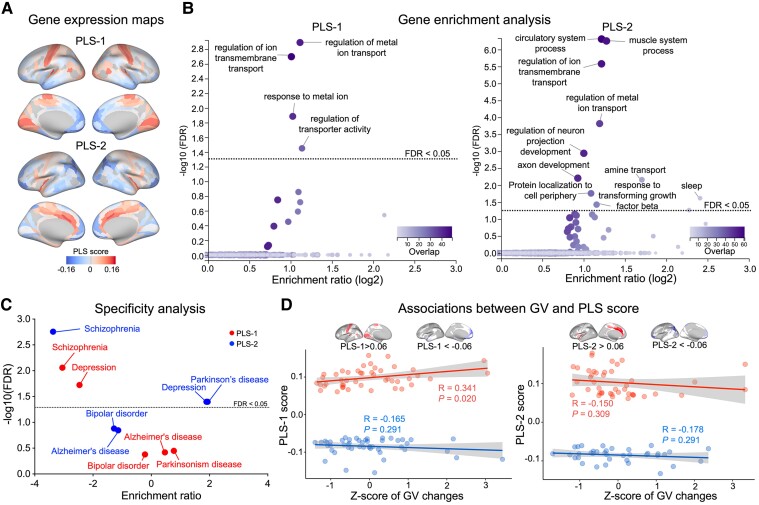
**WD-related gradient alterations associated with gene expression within the cortex.** PLS regression analysis was used to evaluate the association between gradient changes and gene expression. (**A**) Maps of gene expression within the cortex. The color scale indicates the score for PLS-1 and PLS-2, namely, the weighted average expression level of 10 027. (**B**) Gene enrichment analysis. Genes associated with PLS-1 were enriched for ion transport, ion transmembrane transport, and transporter activity, determining metal ion homeostasis, and PLS-2 was enriched for muscle system process and neuron projection development, as well as ion transport and ion transmembrane transport. In the volcano plots, the *x*-axis indicates the log2 ER, and the *y*-axis indicates the −log10 (FDR). The color codes indicate the number of genes related to the biological processes that overlap with the input list of the top 10 percentile genes; the dotted line indicates FDR = 0.05. (**C**) Specificity analysis. Concerning risk genes, PLS-1 and PLS-2 were enriched for genes associated with schizophrenia and depression, while PLS-2 was additionally enriched for those associated with Parkinson’s disease. A bootstrapped permutation test (*N* = 10 000) was used to evaluate the significance of the observed ER, followed by FDR correction for multiple comparisons. The dotted line indicates FDR = 0.05. (**D**) Associations between GV changes and scores >0.06 and <−0.06 for both PLS-1 and PLS-2. A PLS-1 score >0.06 was significantly correlated with GV changes. GV, gray matter volume; PLS, partial least squares.

For the cortex, two PLS components explained 23% (PLS-1, *P* < 0.001) and 21% (PLS-2, *P* < 0.001) of the covariance between the gradient changes in WD and gene expression (Supplementary Fig. 8). PSL-1 represented a gene with high expression mainly in the SMN and VIS but low expression in the LIB and DMN (Fig. 4A). PLS-2 was characterized by high expression in the VAN but low expression in the FPN (Fig. 4A). Gene enrichment analysis revealed that PLS-1 is involved in the regulation of metal ion transport, ion transmembrane transport, and transporter activity (FDR < 0.05, Fig. 4B), which determine metal ion homeostasis. In contrast, PLS-2 mainly reflected muscle system processes, circulatory system, and neuron projection development, as well as the regulation of metal ion transport and ion transmembrane transport (FDR < 0.05, Fig. 4B). For molecular signatures, PLS-1 is involved mainly in transporter activity, whereas PLS-2 is related primarily to molecular function regulators, calcium ion binding, phosphoric ester hydrolase activity and ion gated channel activity (Supplementary Fig. 9). Specificity analysis revealed that PLS-1 and PLS-2 were enriched for risk genes (Fig. 4C), such as genes related to schizophrenia (PLS-1: FDR = 0.0095, ER = -3.05; PLS-2: FDR = 0.0023; ER = −3.37), depression (PLS-1: FDR = 0.0206, ER = −2.46; PLS-2: FDR = 0.044, ER = 1.90), and Parkinson’s disease (PLS-2: FDR = 0.044, ER = 1.94). The changes in GV had a significant positive correlation with PLS-1 scores higher than 0.06. The areas with PLS-1 scores higher than 0.06 and significant changes in GV (Supplementary Fig. 6) exhibited high spatial similarity. Therefore, these findings indicated that structural impairments and gradient perturbations in cortical regions may be mediated by similar transcriptomic specializations (Fig. 4D).

**Figure 5:**
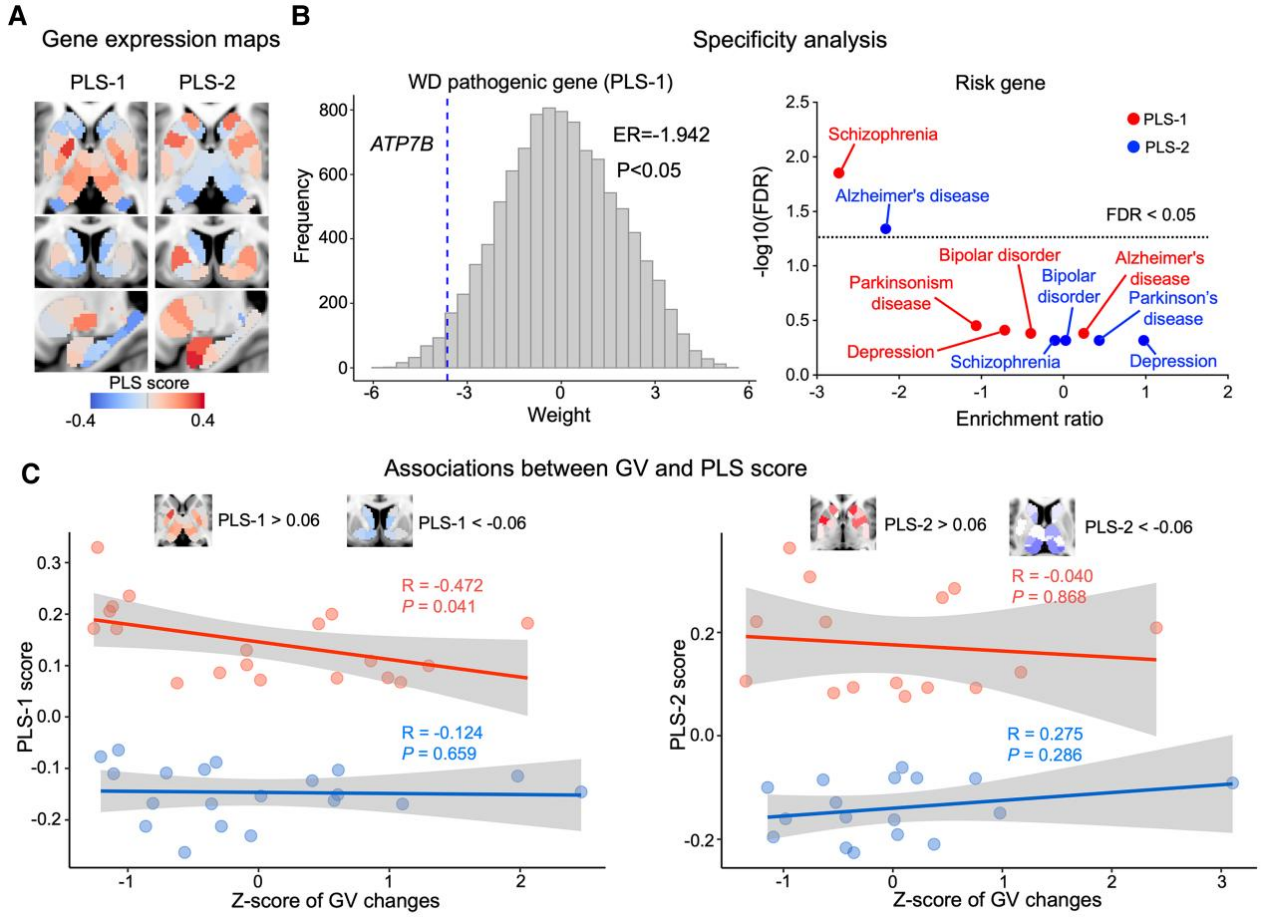
**WD-related gradient alterations associated with gene expression within SUB.** PLS regression analysis was used to evaluate the association between gradient changes and gene expression. (**A**) Maps of gene expression within the SUB. The color scale indicates the score for PLS-1 and PLS-2, namely, the weighted average expression level of 10 027. (**B**) Specificity analysis. PLS-1 was significantly enriched for WD pathogenic genes (*ATP7B*). The histogram shows bootstrap weights of 10 000 permutations, and the blue dotted line indicates the bootstrap weight of the candidate genes. Concerning risk genes, PLS-1 was enriched for genes associated with schizophrenia, while PLS-2 was enriched for those associated with Alzheimer’s disease. A bootstrapped permutation test (*N* = 10 000) was used to evaluate the significance of the observed ER, followed by FDR correction for multiple comparisons. The dotted line indicates FDR = 0.05. (**C**) Associations between GV changes and scores >0.06 and <−0.06 for both PLS-1 and PLS-2. A PLS-1 score >0.06 was significantly correlated with GV changes. GV, gray matter volume; PLS, partial least squares.

For SUB, two PLS components explained 28% (PLS-1, *P* = 0.0264) and 22% (PLS-2, *P* = 0.0437) of the covariance between the gradient changes in WD and gene expression (Supplementary Fig. 8). PSL-1 represented a gene expression profile with high expression mainly in the pallidum and thalamus but low expression in the hippocampus and striatum (Fig. 5A). PSL-2 exhibited high gene expression in the pallidum, striatum, and amygdala but low expression mainly in the thalamus (Fig. 5A). Specificity analysis revealed that PLS-1 (Fig. 5B) was enriched for the risk gene of WD (PLS-1: *P* = 0.022, ER = −1.946), with PLS-1 and PLS-2 additionally enriched for the genes causing schizophrenia (PLS-1: FDR = 0.0155, ER = −2.73) and Alzheimer’s disease (PLS-2: FDR = 0.0425, ER = −2.16). Additionally, GV changes had a significant negative correlation with PLS-1 scores greater than 0.06, indicating that structural impairments and gradient perturbations in SUB were also mediated by similar transcriptomic specializations (Fig. 5C).

## Discussion

Macroscale hierarchy has been widely regarded as a key principle of brain organization that spans from primary to transmodal networks.^13^ Hierarchical representations account for the spatial arrangement of local processing streams throughout the cerebral cortex, ensuring that sensory signals are progressively integrated as abstract representations.^17^ According to the hierarchical theory, both WD patients and HCs revealed that principal functional gradient mapping captured the macroscale axis of connectivity variations, with the primary network and transmodal DMN anchored at two opposite ends and the remaining networks emerging between them.^17^ Since WD has been clinically characterized as a disorder with multiple neurological and psychiatric phenotypes,^35^ it may result in errors in information flow from sensorimotor to cognitive processing. Therefore, the principal gradient of WD was globally suppressed at both ends compared with that of HC in the present study. Notably, VBM analysis also illustrated that in WD, GV significantly decreased in multiple systems, including the SMN, VIS, and transmodal areas. Further spatial correlations between the gradient and GV revealed structure–function decoupling in the VIS, VAN, and SUB, suggesting that hierarchical imbalances might be derived from structural alterations in the WD.

At the regional level, patients with WD exhibited increased gradient scores in primary systems such as the SMA and V1 and decreased scores in transmodal regions, including the mTP and mPFC. Consistent with the present study, WD-related abnormalities in intrinsic brain activity in the SMN and VN have been highlighted in RS-fMRI^8^ and tomography-electrophysiology studies.^36^ Generally, an important role of the SMA and V1 in motor control and visual processing has been identified in the motor and visual systems, particularly for postural stabilization of the body and spatial recognition.^37-40^ Moreover, cortical thickness damage in these regions was also reported to be correlated with neurological symptoms in WD patients.^41^ Thus, these findings indicated that gradient imbalances in the SMA and VN contribute to neurological symptoms in WD patients, such as dyskinesia. On the other hand, DMN areas, such as the mPFC and mTP, are thought to be involved in several different functions, such as self-referential and introspective cognition as well as mentalizing.^42,43^ Dysfunction in these regions may lead to neuropsychological and attention network deficits in patients with WD.^9,44^ Thus, gradient perturbations in the DMN may result in neuropsychological symptoms in WD patients. Notably, our meta-analysis revealed that increased WD-related gradient scores are primarily involved in low-level sensory and motor-related processes, while decreased gradient scores are more strongly associated with higher-order cognitive function. Thus, these findings suggested that the imbalance in the functional hierarchy might arise from the disorder of local processing streams from primary to transmodal systems within the cerebral cortex of WD patients. In addition, our results also showed increased gradient scores in SUB, such as in the putamen and thalamus, in WD patients. Due to the impaired regions leading to disrupted networks,^16^ the interactions among SUB and between subcortical-cortical networks could impact the topology of the subcortical macroscale connectome in WD patients. Furthermore, recent studies have reported that connectivity among SUB was strongly associated with deficits in neurological symptoms,^45^ and the subcortical-cortical pathways were related to neuropsychological disorders in WD patients.^9^ In this context, the relationships between the primary gradients and UWDRS-N were mediated by GV alteration, indicating that structural vulnerability impacts the macroscale hierarchy and ultimately leads to deficits in the WD phenotype.

We identified the spatial association between the perturbations in the connectome gradient and gene expression profiles to determine the transcriptomic specialization of WD. Within the cortex, PLS-1 mediated the regulation of ion transmembrane transport, metal ion transport, and transporter activity. In biology, the transporter is a transmembrane protein that moves ions across biological membranes to accomplish different biological functions, such as cellular communication and maintaining homeostasis.^46^ Ion transport is a biological process in which ions are transported along a concentration gradient from high to low concentrations.^47^ Thus, these findings may reflect the biological mechanism of abnormal copper ion metabolism in WD patients. Moreover, PLS-2 within the cortex also mediated the regulation of ion transmembrane transport and ion gated channel activity but more strongly reflected the regulation of neuron projection development and axon development, as well as a muscle system process. Neuronal projection is any process extending from a neural cell, such as axons and dendrites. Its development progresses from neural formation to mature structure^48^ and promotes neuron differentiation.^49^ The muscle system in vertebrates is controlled through the nervous system, and its impairment leads to different movement disorders.^50^ Indeed, WD is a disorder of copper transport within the cell resulting in copper accumulation in the brain,^51^ which may trigger abnormal degeneration of neurons. Therefore, PLS-1 likely represents biological mechanisms underlying ion homeostasis inside and outside nerve cells, while PLS-2 was more strongly associated with neural organization and cell growth as well as controlling muscle signaling. Furthermore, transcriptomic specilizations of WD have also been reported to be associated with risk genes for schizophrenia, depression and Parkinson’s disease, which may explain why WD patients present multiple complex neurological and psychiatric symptoms at the biological level.

Within SUB, we demonstrated that PLS-1 was associated with the WD pathogenic gene, which indicates that the *ATP7B* gene has lower expression in SUB. Thus, for the first time, we provided evidence that WD pathogenic genes impact subcortical function in WD patients. Within SUB, transcriptomic specilization identified by PLS-1 and PLS-2 was associated with risk genes for schizophrenia and Alzheimer’s disease. Consistent with our results, recent studies have reported that the risk genes for schizophrenia^52^ and Alzheimer’s disease^53^ are significantly associated with subcortical dysfunction. Therefore, these findings demonstrated that functional gradient perturbations in SUB also contribute to complex neurological or psychiatric symptoms. Notably, we found that the spatial pattern of GV alterations is negatively related to that of PLS-1 score in SUB. However, GV alterations had a positive spatial correlation with PLS-1 in the cortex. This might be attributed to differences in gene expression in the cerebral cortex and SUB.^54^ Collectively, the present findings indicate that GV impairments and gradient perturbations might be shapped by similar transcriptomic specializations.

The findings must be interpreted in the context of potential limitations. First, gene sets from AHBA were sampled from a small sample of donors. Therefore, in the PLS analysis, the associations between the connectome gradient and gene expression were susceptible to intersubject variability. On the other hand, all the gene samples were not from the brains of patients with WD. Therefore, the spatial correlation between gradient changes and transcriptomic data cannot directly reflect how connectome gradient purturbation is affected by transcriptional abnormalities in WD. In the future, a larger sample of whole-brain gene expression data from WD patients is required to represent and validate the relationship between the connectome gradient and transcriptome. Second, the difference in age between the two groups was significant. Although we used age as a covariate in the statistical analysis, we cannot completely eliminate the effect of age on the results. Therefore, in future studies, we will recruit age- and sex-matched healthy subjects to study structural and functional abnormalities in WD patients and their relationships with gene expression. Finally, long-term copper deficiency leading to structural changes in the brain has been reported in a previous study,^55^ which may also result in brain damages in individuals. In future studies, adding patients with copper deficiency phenotype as a comparison will be of great significance to further understand the neural mechanism of WD.

This study revealed gradient perturbations related to cognitive terms and clinical phenotypes in WD patients. Then, we evaluated the associations of the gradient perturbation with specific GV alterations and gene expression profiles. Furthermore, we revealed the biological underpinnings of these gradient-derived gene sets by enrichment analysis. The biological underpinnings were determined by underlying factors, including (1) structure–function is decoupled in WD; (2) strong associations between gene expression and gradient differences have identified transcriptomic specializations within both the cortex and SUB of WD; (3) gradient perturbations are implicated in psychiatric and neurological diseases and for the first time characterized the role of *ATP7B* in subcortical function; and (4) GV impairments and gradient perturbations were mediated by similar transcriptomic specializations. In summary, these findings reveal the structural and transcriptomic underpinnings of gradient perturbations in WD, providing deep insight into the neurobiological mechanisms underlying the emergence of complex neurological and psychiatric phenotypes in WD patients.

## Supplementary Material

fcae329_Supplementary_Data

## Data Availability

The data that support the findings of this study are available from the corresponding author upon reasonable request. The gene expression data used for transcriptional analysis can be found in the ABHA database (https://human.brain-map.org/static/download).
