# Supplementary material for: Structural lesions and transcriptomic specializations shape gradient perturbations in Wilson disease
Source: Brain Commun. 2024 Sep 24;6(5):fcae329. doi: 10.1093/braincomms/fcae329 (PMC11450269; doi:10.1093/braincomms/fcae329)
Supplement: fcae329_Supplementary_Data [file fcae329_supplementary_data.docx]

**Supporting information for**

**Structural lesions and transcriptomic specializations shape gradient perturbation in Wilson disease**

Sheng Hu^1,3,4^, Chuanfu Li^5^, Yanming Wang^1^, Taohua Wei^2,6^, Xiaoxiao Wang^1^, Ting Dong^2,6^, Yulong Yang^2,6^, Yufeng Ding^2,6^, Bensheng Qiu^1,3^, Wenming Yang^2,6^

**Author affiliations:**

1 Medical Imaging Center, Department of Electronic Engineering and Information Science, University of Science and Technology of China, Hefei, Anhui, 230026, China

2 Department of Neurology, First Affiliated Hospital of Anhui University of Traditional Chinese Medicine; Hefei, Anhui, 230031, China

3 Institute of Advanced Technology, University of Science and Technology of China; Hefei, Anhui, 230094, China

4 School of Medical Information Engineering, Anhui University of Traditional Chinese Medicine; Hefei, Anhui, 230012, China

5 Medical Imaging Center, First Affiliated Hospital of Anhui University of Traditional Chinese Medicine, Hefei, Anhui, 230031, China

6 Key Laboratory of Xinan Medicine of the Ministry of Education, Anhui University of Traditional Chinese Medicine, Hefei, Anhui, 230031, China

Correspondence to: Wenming Yang

Department of Neurology, The First Affiliated Hospital of Anhui University of Chinese Medicine, Hefei, Anhui, 230031, China

E-mail: [yangwm8810@126.com](mailto:yangwm8810@126.com)

Correspondence may also be addressed to: Bensheng Qiu

Center for Biomedical Imaging, University of Science and Technology of China; Hefei, Anhui, 230026, China

E-mail: [bqiu@ustc.edu.cn](mailto:bqiu@ustc.edu.cn)

**Running title**: Structural and transcriptional marks of WD

**Summary:**

1 of 1 Supplementary Methods

9 of 9 Supplementary Color Figures

7 of 7 Supplementary Tables

# Supplementary Methods

## fMRI data preprocessing

Preprocessing of the functional images involved the following steps: (1) discarding the first 10 time points of functional images to account for magnetic stabilization; (2) slice timing correction to compensate for temporal shifts of different slices; (3) within-subject fMRI image realignment to estimate and spatially correct for head motions of different volumes; (4) rigid-body T1 image registration to the functional mean image and functional image normalization to the Montreal Neurological Institute (MNI) standard space using the T1 images; (5) functional image resampling to 3×3×3 mm^3^ voxel size followed by regressing out confounding signals, including linear trends, white matter (WM), cerebrospinal fluid (CSF), and Friston’s 24 head motion parameters; (6) bandpass filtering (0.01-0.1 Hz) to suppress low-frequency drift and physiological noises (breathing and heartbeat); and (7) functional image spatial smoothing using a 6 mm full-width at half-maximum (FWHM) Gaussian kernel. All data preprocessing was performed on DPABI version 5.3 (http://rfmri.org/dpabi), a MATLAB toolbox for batch fMRI data preprocessing.

# Supplementary Figures


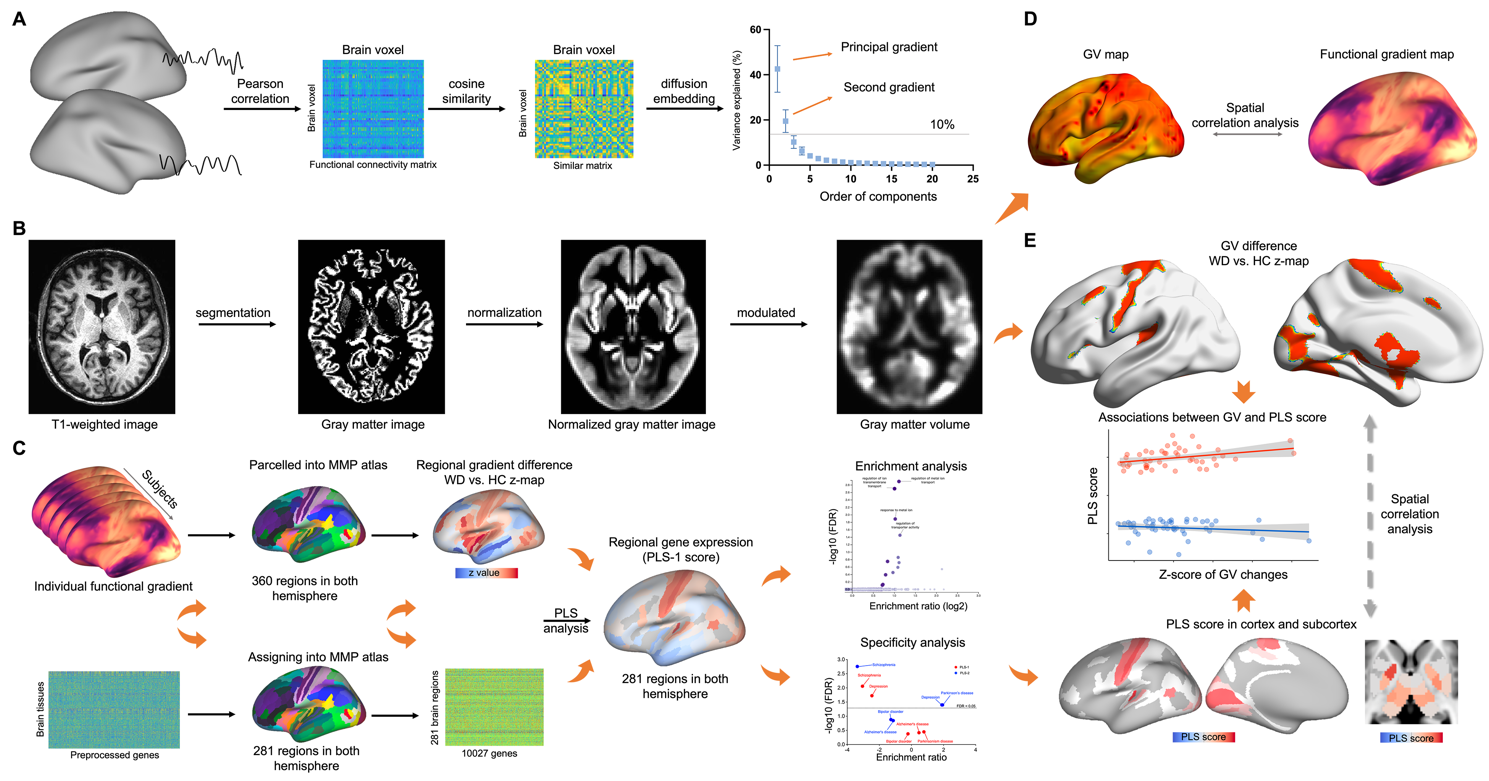


**Supplementary Figure 1 Study overview. (A)** Functional gradient analysis. A functional connectivity (FC) matrix was first constructed by performing Pearson correlation analysis between each pair of gray matter nodes (20208 voxels). Then, the FC matrix was further applied to the diffusion map embedding method to calculate the connectome gradient. **(B)** The gray matter was first segmented from the T1-weighted images, followed by normalization to MNI152 standard space. The normalized images were further applied to create a study-specific GM template. All native GM images were subsequently registered to the template and modulated for contraction. **(C)** The functional gradient and gene expression were first projected to cortical atlas with 360 parcels, then PLS regression analysis was performed to evaluate relationships between functional gradient difference and gene expression, and finally enrichment analysis and specificity analysis were conducted to determine the biological processing of gradient perturbation in WD. The flowchart of association analysis between functional gradient difference with subcortical regions and gene expression was same with that within the cortex. **(D)** The spatial correlation analysis between GV map and functional gradient map was performed to unravel the struncture-function coupling for both WD and HC. **(E)** The relationships between GV difference and PLS score was evaluated using spatial correlation analysis. WD, Wilson’s disease; HC, healthy control; GV, gray matter volume; PLS, partial least squares.


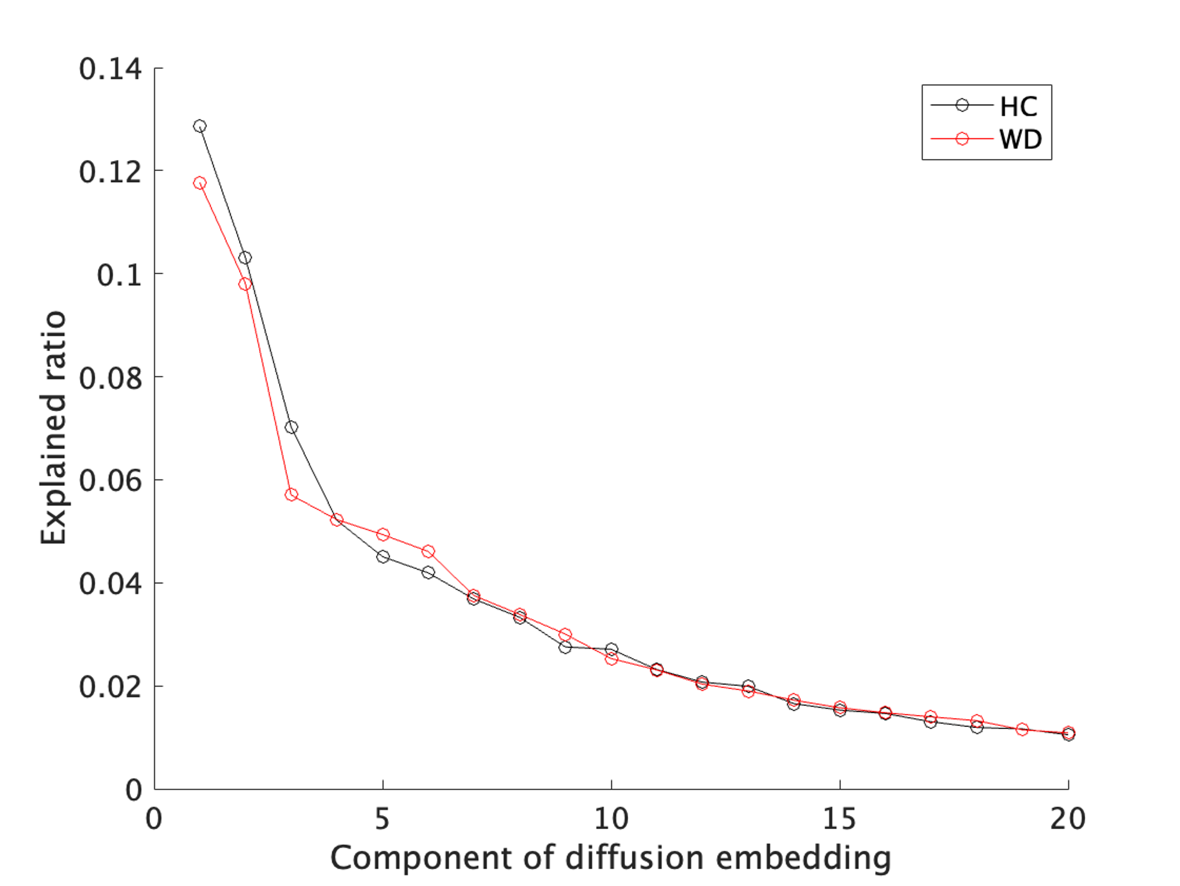


**Supplementary Figure 2** **The averaged explained ratio** (λ values). The first two gradients explained 22.2 ± 2.9% of the total variance in the connectome across all individuals (WD: 21.6 ± 2.9%, HC: 23.2 ± 2.8%). WD, Wilson’s disease; HC, healthy control.


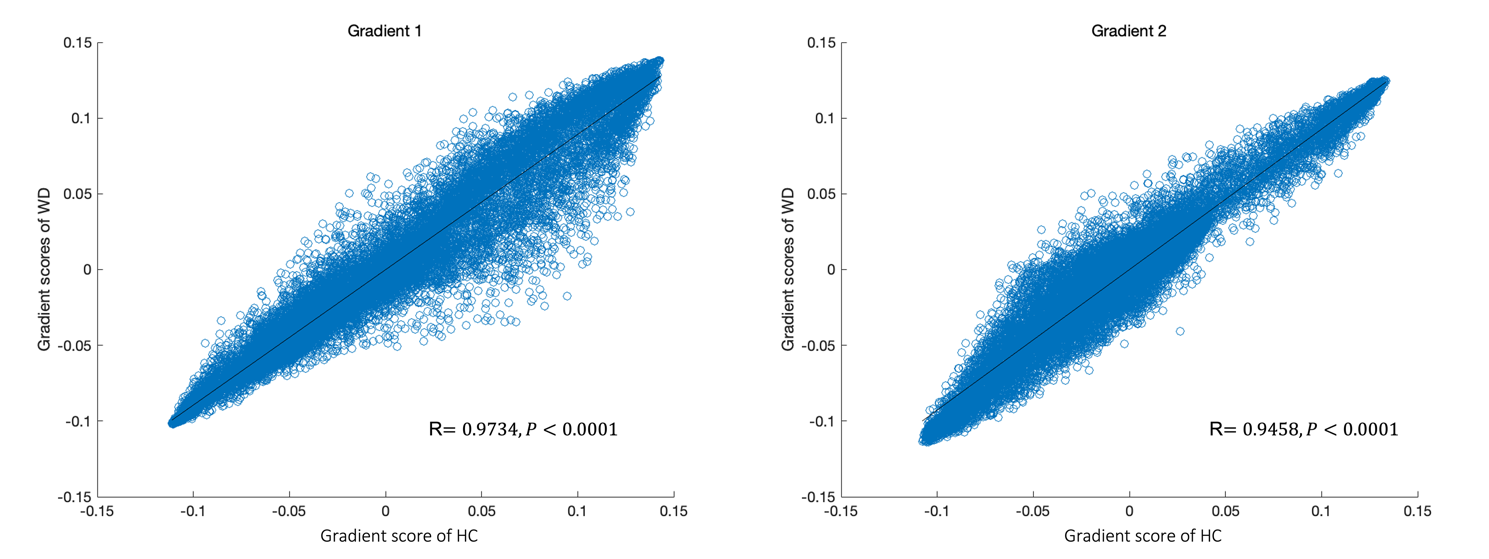


**Supplementary Figure 3** **Spatial correlations of the group-averaged gradient maps between the WD and HC.** These correlations were corrected for spatial autocorrelations by using a permutation test (N = 10,000). WD, Wilson’s disease; HC, healthy control.


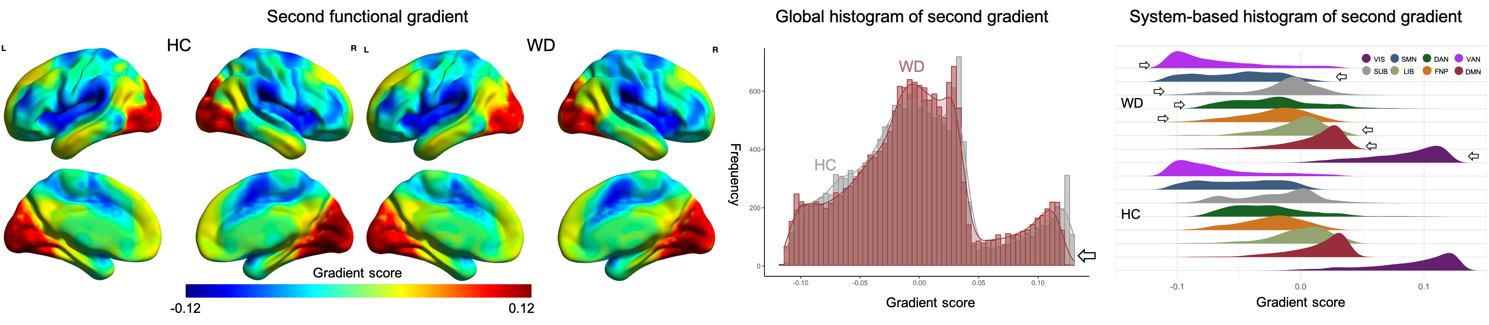


**Supplementary Figure 4** **Second connectome gradient.** The group-level connectome gradient mapping of both WD (n = 105) and HC (n = 93) were calculated from group mean functional connectivity matrix. Secondary gradient mapping in patients with MDD and controls (left). The global (middle) and system-based histograms (right) show that the extreme values in the patients with WD were contracted relative to those in the HC in the secondary gradient. The system-based differences of second gradient distribution between WD and HC were evaluated using two-sample t test with age, sex, and education as covariates. A two-tailed *P* < 0.05 after FDR correction for multiple comparisons was considered significant. VIS, visual network; SMN, sensorimotor network; DAN, dorsal attention network; VAN, ventral attention network; SUB, subcortical regions; LIB, limbic network; FPN, frontoparietal network; DMN, default mode network. WD, Wilson’s disease; HC, healthy control.


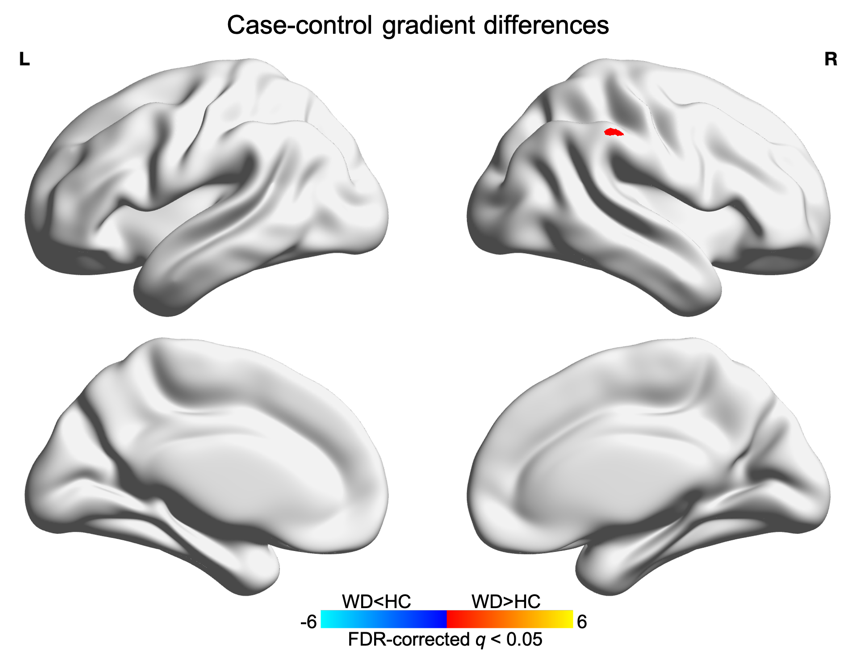


**Supplementary Figure 5 Statistical comparison of the secondary gradients between the WD and HC.** Two-sample t test was performed to evaluate group difference with age, sex, and education as covariates. WD, Wilson’s disease; HC, healthy control.


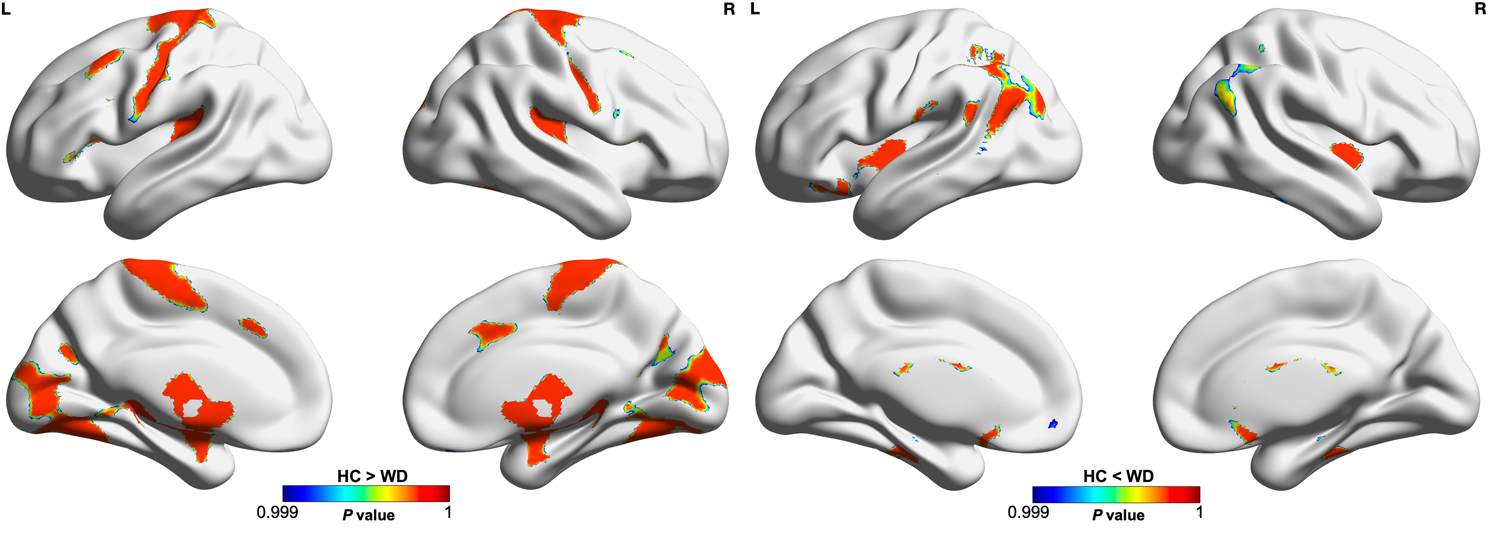


**Supplementary Figure 6 The group differences of gray matter volume between WD and HC.** The statistical significance was set as P < 0.001, using a threshold-free cluster enhancement approach with family wise-error (FWE) correction for multiple comparisons. WD, Wilson’s disease; HC, healthy control.


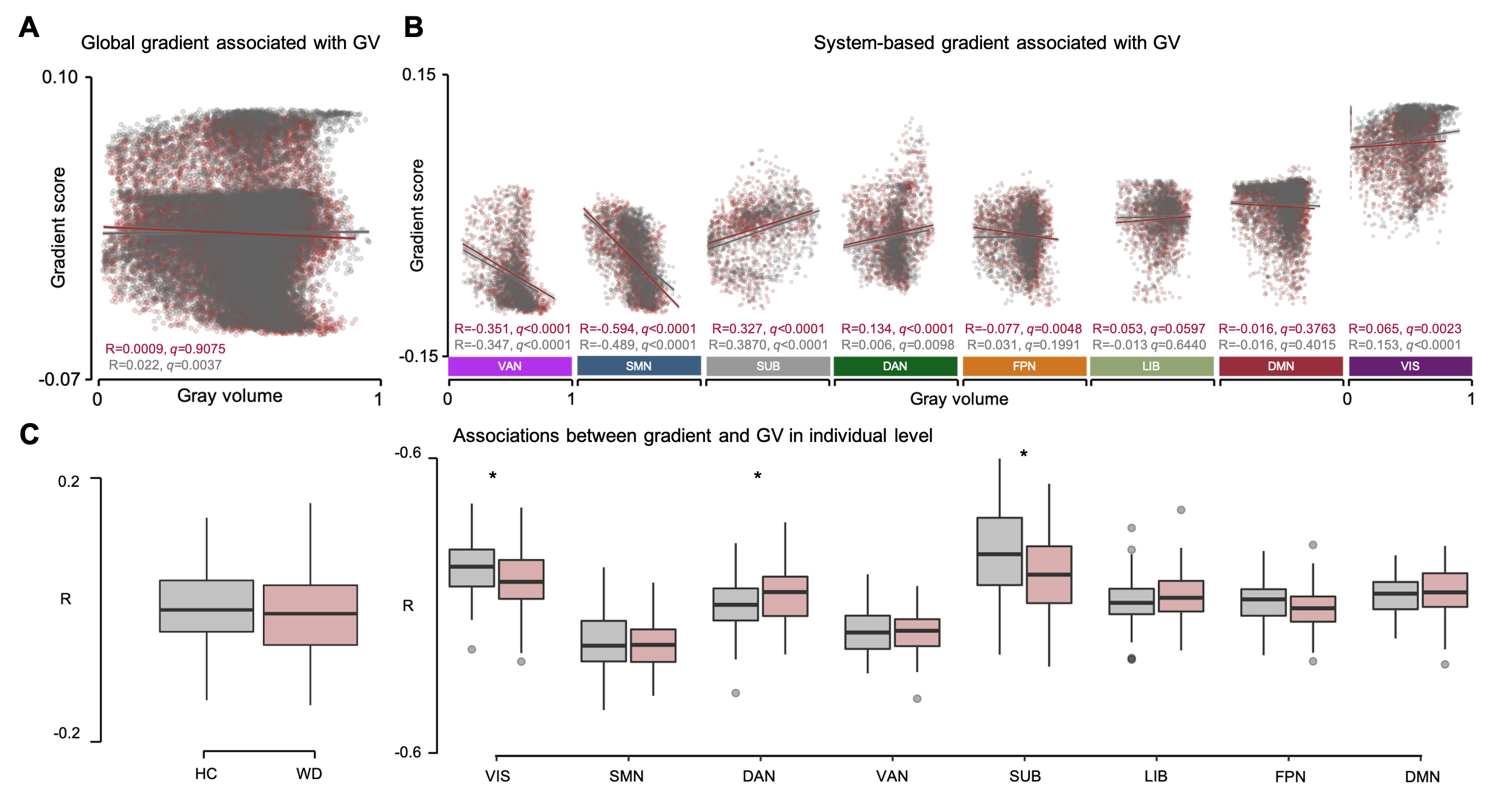


**Supplementary Figure 7** **Spatial correlations between secondary gradient and GM volume.** (**A)** Spatial correlation between global secondary gradient and GM volume. **(B)** System-based spatial correlations between secondary gradient and GM volume. **(C)** Global and system-based associations between gradient and GV at the individual level and their group differences between WD (n = 105) and HC (n = 93) were measured by a two-sample t test and corrected by FDR. *, *P* < 0.05. VIS, visual network; SMN, sensorimotor network; DAN, dorsal attention network; VAN, ventral attention network; SUB, subcortical regions; LIB, limbic network; FPN, frontoparietal network; DMN, default mode network. WD, Wilson’s disease; HC, healthy control; GV, gray matter volume.

**
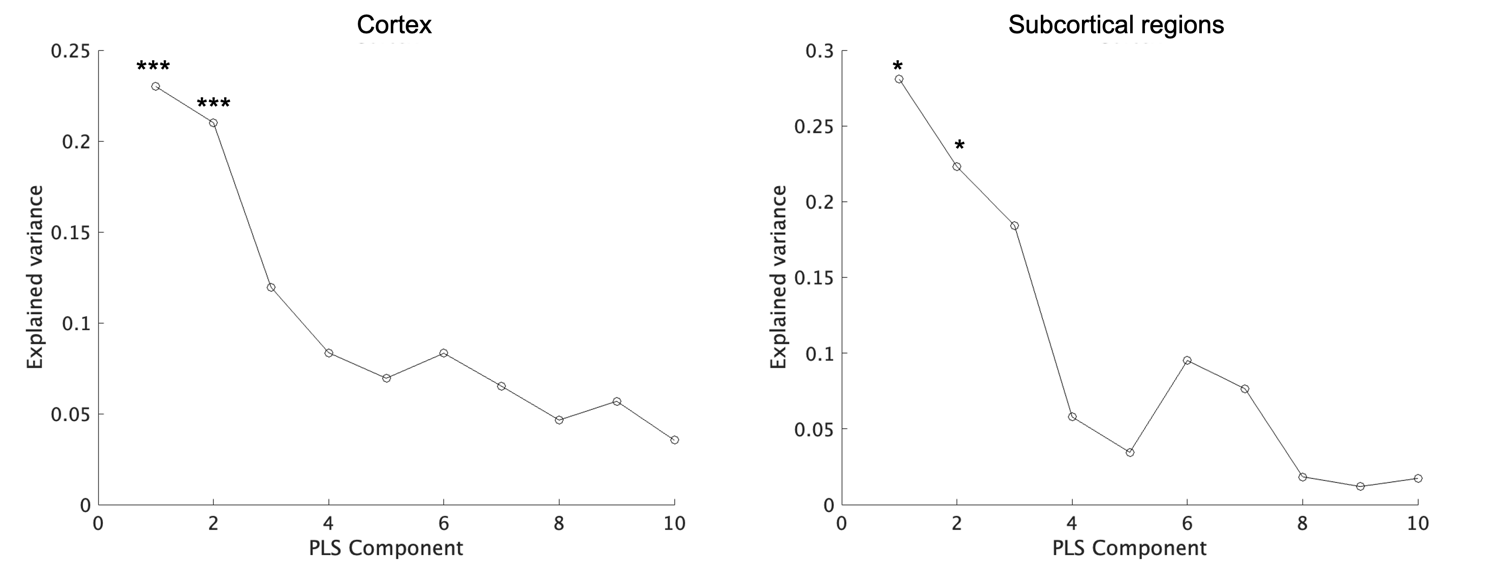
**

**Supplementary Figure 8** **The percentage of variance in the response variables explained by the components in the partial least square regression analysis.** The significance level was determined by a permutation test (N = 10,000) with spatial autocorrelation corrected. ***, *P* <0 .001; *, *P* < 0.05. PLS, partial least squares.


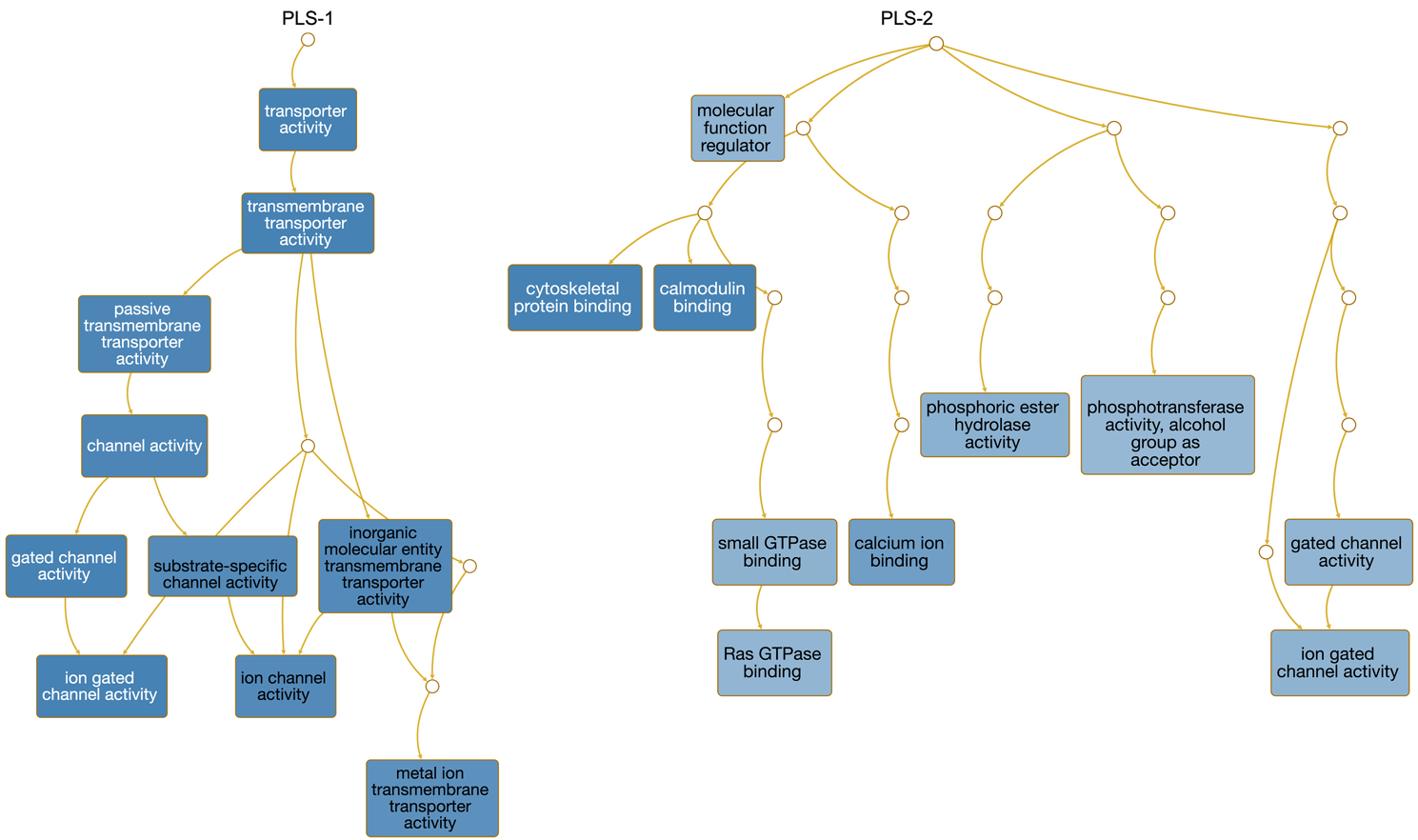


**Supplementary Figure 9 Gene enrichment analysis for identifying molecular function of gradient perturbation.** PLS-1 mainly involves in transporter activity, whereas PLS-2 primarily relates to molecular function regulator, calcium ion binding, phosphoric ester hydrolase activity and ion gated channel activity.

# Supplementary Tables

**Supplementary Table 1** The lists of candidate genes used for disease specificity analysis are presented in Fig. 4 and 5.

| A | *A2M, ACE, ACHE, APBA1, APBB2, APLP1, APLP2, APOC1, APP, BACE2, BCHE, BLMH, CASP3, CHRNA3, CTSB, DBN1, ESR1, GSK3B, IL1B, KCNIP3, KLK6, LRP1, LRRC15, MAPT, PLAU, PSEN1, PSEN2, SORL1* |
| --- | --- |
| B | *ATP1A3, DBH, DDC, HTRA2, LRRK2, NR4A2, PARK2, PARK7, PINK1, SLC6A3, SNCA, SNCAIP, SNCB, TH, UCHL1* |
| C | *ACTN2, AHI1, AKT1, ANKHD1, APOL1, APOL2, APOL4, ATF4, ATF5, ATF7IP, BDNF, CCDC141, CHRM1, CHRNA7, CIT, COMT, DAO, DAOA, DISC1, DRD1, DRD3, DRD5, DTNBP1, ERBB3, ERBB4, FEZ1, GABRA6, GABRB2, GAD1, GRIN1, GRIN2A, GRIN2B, GRM2, GRM3, GRM7, HOMER1, HTR2A, HTR2C, HTR6, ITSN1, KCNN3, MAP1A, NDE1, NDEL1, NPAS3, NRG1, PAFAH1B1, PCNT, PDE4B, PDLIM5, PLXNA2, PPP1R1B, PPP3CA, PPP3CC, PRODH, PVALB, RANBP9, RASSF7, RGS4, RTN4, RTN4R, SLC17A7, SPTBN4, SYN2, SYNE1, TAAR6, TNF* |
| D | *ADRA2A, AVPR1B, CHRM2, CNR1, CREB1, CRH, CRHR1, CRHR2, CUX2, GAD2, GPR50, HTR1A, HTR1B, HTR1D, HTR3A, HTR5A, MAOA, PDE1A, SLC6A2, SLC6A4, SST, TAC1, TPH1, TPH2* |
| E | *ADRBK2, CLOCK, XBP1* |

1. Alzheimer’s disease, (B) Parkinson‘s disease, (C) Schizophrenia, (D) Depression, (E) Bipolar disease

**Supplementary Table 2** Between-group differences in the principal gradient scores of the subnetworks in the mean maps

| Subnetworks | *t* | Cohen’s *d* | *P* | *FDR q* |
| --- | --- | --- | --- | --- |
| Visual network | 38.475 | 0.777 | <1.0×10^-20^ | <1.0×10^-20^ |
| Sensorimotor network | 45.071 | 0.970 | <1.0×10^-20^ | <1.0×10^-20^ |
| Dorsal attention network | 1.889 | 0.046 | 0.0589 | 0.0589 |
| Ventral attention network | 20.223 | 0.523 | <1.0×10^-20^ | <1.0×10^-20^ |
| Limbic network | -25.348 | 0.699 | <1.0×10^-20^ | <1.0×10^-20^ |
| Frontoparietal network | 9.3046 | -0.201 | 3.221×10^-20^ | 4.294×10^-20^ |
| Default mode network | -29.036 | -0.503 | <1.0×10^-20^ | <1.0×10^-20^ |
| Subcortical network | 8.032 | 0.254 | 2.685×10^-15^ | 3.069×10^-15^ |

Abbreviations: FDR, false discovery rate.

**Supplementary Table 3** Clusters with significant between-group differences in the principal primary-to-transmodal gradient

| No. | Region | x | y | z | Z (Peak) | Cohen’s *d* | Size (voxel) |
| --- | --- | --- | --- | --- | --- | --- | --- |
| WD > HC | |  |  |  |  |  |  |
| 1 | Right SMA | 2 | -10 | 60 | 4.91 | 0.13 | 128 |
| 2 | Right primary visual cortex | 10 | -86 | 8 | 4.27 | 0.10 | 126 |
| 3 | Left putamen | -18 | 14 | -4 | 5.58 | 0.17 | 61 |
| 4 | Left insular lobe | -34 | 22 | 8 | 4.64 | 0.12 | 57 |
| 5 | Right putamen | 22 | 10 | -4 | 5.81 | 0.18 | 49 |
| 6 | Left middle frontal gyrus | -26 | 38 | 24 | 4.94 | 0.13 | 42 |
| 7 | Right inferior temporal gyrus | 50 | -62 | -8 | 3.87 | 0.08 | 35 |
| WD < HC | |  |  |  |  |  |  |
| 8 | Right medial temporal pole | 38 | 14 | -40 | -5.75 | -0.18 | 217 |
| 9 | Left temporal pole | -34 | 18 | -24 | -5.47 | -0.16 | 210 |
| 10 | Left superior frontal gyrus | -22 | 46 | 44 | -5.22 | -0.15 | 160 |
| 11 | Right medial prefrontal cortex | 10 | 50 | 48 | -5.83 | -0.18 | 139 |
| 12 | Left rectal gyrus | -6 | 54 | -24 | -5.92 | -0.19 | 52 |

**Supplementary Table 4** Between-group differences in the secondary gradient scores of the subnetworks in the mean maps

| Subnetworks | *t* | Cohen’s *d* | *P* | *FDR q* |
| --- | --- | --- | --- | --- |
| Visual network | -29.634 | -0.598 | <1.0×10^-20^ | <1.0×10^-20^ |
| Sensorimotor network | -7.230 | -0.156 | 6.674×10^-13^ | 7.628×10^-13^ |
| Dorsal attention network | 10.824 | 0.265 | <1.0×10^-20^ | <1.0×10^-20^ |
| Ventral attention network | 11.796 | 0.305 | <1.0×10^-20^ | <1.0×10^-20^ |
| Limbic network | -9.702 | -0.267 | <1.0×10^-20^ | <1.0×10^-20^ |
| Frontoparietal network | 12.446 | 0.269 | 3.221×10^-20^ | 4.294×10^-20^ |
| Default mode network | -19.399 | -0.336 | <1.0×10^-20^ | <1.0×10^-20^ |
| Subcortical network | 4.758 | 0.151 | 2.241×10^-6^ | 2.241×10^-6^ |

Abbreviations: FDR, false discovery rate.

**Supplementary Table 5** Clusters with significant between-group differences in the secondary gradient

| No. | Region | x | y | z | *Z* | Cohen’s *d* | Size (voxel) |
| --- | --- | --- | --- | --- | --- | --- | --- |
| WD > HC | |  |  |  |  |  |  |
| 1 | Right supramarginal gyrus | 66 | -30 | 44 | 6.00 | 0.13 | 7 |

**Supplementary Table 6** Spatial correlation between the meta-analytic map of cognitive terms and WD-related alterations in the primary gradient

| WD-positive | |  | WD-negative | | |  |
| --- | --- | --- | --- | --- | --- | --- |
| Term | | *r* | | Term | | *r* |
| motor | 0.253 | | | | theory mind | 0.258 |
| somatosensory | 0.228 | | | | mind | 0.24 |
| movement | 0.216 | | | | social | 0.235 |
| movements | 0.201 | | | | mental states | 0.231 |
| pain | 0.196 | | | | mind tom | 0.222 |
| motor imagery | 0.189 | | | | mentalizing | 0.207 |
| hand | 0.196 | | | | theory | 0.187 |
| painful | 0.188 | | | | person | 0.182 |
| execution | 0.186 | | | | autobiographical memory | 0.152 |
| finger | 0.165 | | | | beliefs | 0.135 |
| visual | 0.154 | | | | construction | 0.123 |
| gain | 0.14 | | | | episodic | 0.121 |
| imagery | 0.139 | | | | social cognition | 0.121 |
| foot | 0.138 | | | | memories | 0.12 |
| primary visual | 0.136 | | | | dementia | 0.118 |
| early visual | 0.135 | | | | self-referential | 0.117 |
| action | 0.131 | | | | thinking | 0.094 |
| preparation | 0.129 | | | | memory retrieval | 0.094 |
| sighted | 0.123 | | | | comprehension | 0.093 |
| extrastriate | 0.122 | | | | episodic memory | 0.091 |
| finger movements | 0.116 | | | | semantic memory | 0.091 |
| vision | 0.116 | | | | social interaction | 0.085 |
| motor task | 0.116 | | | | intentions | 0.082 |
| sensory | 0.115 | | | | experiences | 0.081 |
| actions | 0.111 | | | | rest | 0.069 |
| tapping | 0.11 | | | | sentence | 0.068 |
| executed | 0.108 | | | | read | 0.059 |
| mirror | 0.107 | | | | concrete | 0.057 |
| force | 0.095 | | | | reasoning | 0.057 |
| stimulation | 0.094 | | | | judgments | 0.054 |

**Supplementary Table 7** Clusters with significant between-group differences in gray matter volume

| Subnetworks | WD < HC | | WD > HC | |
| --- | --- | --- | --- | --- |
|  | *P* (Mean) | Voxel size | *P* (Mean) | *Voxel size* |
| Visual network | 0.9997 | 5283 | 0.9997 | 747 |
| Sensorimotor network | 0.9997 | 6793 | 0.9997 | 450 |
| Dorsal attention network | 0.9996 | 1273 | 0.9997 | 1676 |
| Ventral attention network | 0.9996 | 1250 | 0.9997 | 1928 |
| Limbic network | 0.9993 | 184 | 0.9997 | 1523 |
| Frontoparietal network | 0.9996 | 1010 | 0.9997 | 1572 |
| Default mode network | 0.9996 | 5283 | 0.9997 | 747 |
| Subcortical network | 0.9998 | 4434 | 0.9997 | 269 |
